# Anti-influenza T cells in bronchoalveolar microenvironment of critically severe COVID-19 patients

**DOI:** 10.1186/s13054-021-03871-4

**Published:** 2021-12-20

**Authors:** Ming Zheng

**Affiliations:** 1 Institute of Military Cognition and Brain Sciences, Academy of Military Medical Sciences, 27 Taiping Road, Beijing, 100850 China; 2 Beijing Institute of Basic Medical Sciences, 27 Taiping Road, Beijing, 100850 China

Until December 13, 2021, there have been 269,468,311 confirmed infection cases and 5,304,248 deaths attributed to COVID-19 caused by SARS-COV-2 virus (https://covid19.who.int/). Recently, the global COVID-19 pandemic is merging with the influenza season, leading to an increased risk of COVID-19 and influenza co-infection. The rates of COVID-19 and influenza co-infection were reported to range from 0.2 to 45.7% in different COVID-19 cohorts [1]. Moreover, both SARS-CoV-2 and influenza viruses preferentially infect alveolar type 2 (AT2) cells [2]. The co-infection with influenza A virus causes more severe and prolonged pneumonia in SARS-CoV-2-infected hamsters [3]. However, there is still a lack of evidence to support the co-infection of SARS-CoV-2 with influenza virus in the bronchoalveolar microenvironment of human lungs, and T cell response under this circumstance remains unknown.

The specificity of T cell response is presented by the antigen-specific T cell clonotypes defined by their unique T cell receptors (TCRs) [4]. TCR directly recognizes and interacts with the antigenic epitope, which determines the specificity of T cell response [4]. Thus, the antigenic T cell response can be directly analyzed by epitope-specific TCR sequence [4]. Recently, T-Detect™ COVID (Adaptive Biotechnologies)—the first assay of detecting TCR-β chain—has been approved by the US Food and Drug Administration (FDA) to determine SARS-CoV-2 infection status based on SARS-CoV-2 specific TCR-β sequence [5].

Here, we constructed the analysis framework of digitalized sorting of epitope-specific T cells (DSET; Fig. 1A) according to the amino acid sequence of complementarity determining region 3 (CDR3) in TCR-β, as reported in our previous study [4]. The marker of influenza infection—TCR-β CDR3 sequences of influenza A-specific T cell clonotypes—was collected from previous studies (Fig. 1A). Next, through applying DSET analysis on the single-cell T cell receptor sequencing (scTCR-seq) data of bronchoalveolar lavage fluid (BALF) in a COVID-19 cohort [6], we directly investigated the anti-influenza T cells in bronchoalveolar microenvironment of COVID-19 patients.

**Fig. 1:**
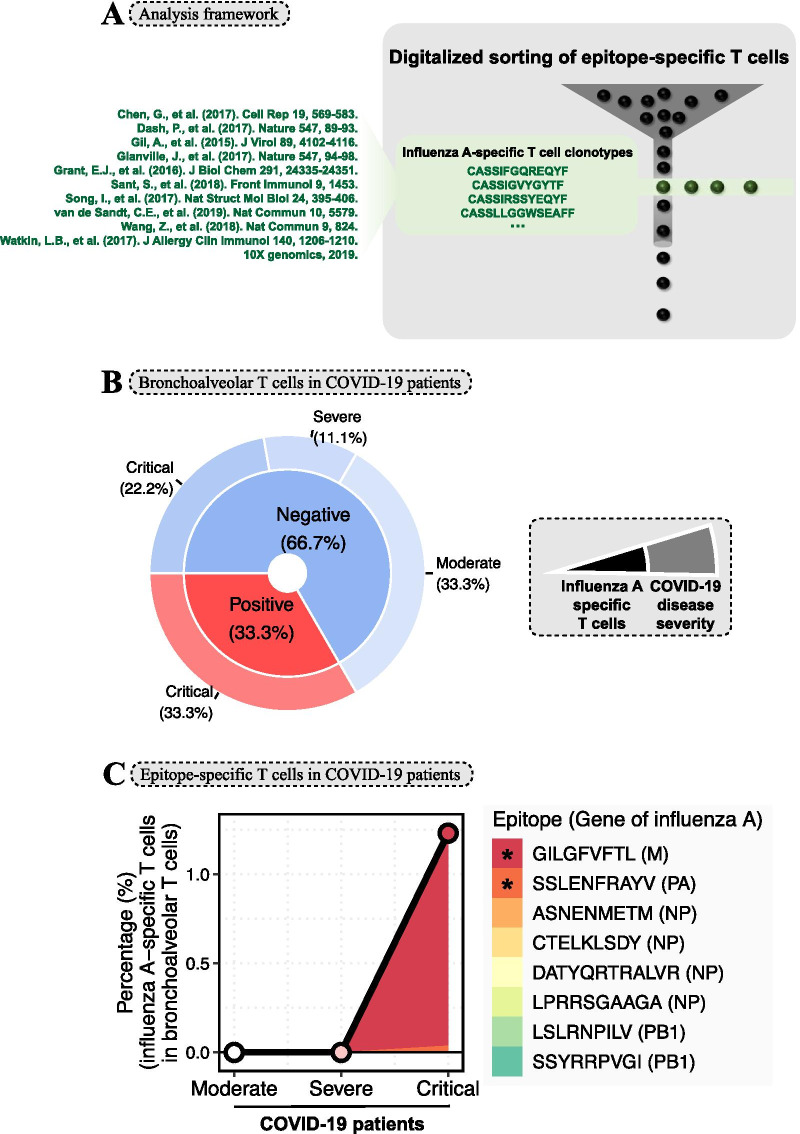
Influenza A-specific T cell clonotypes in the bronchoalveolar lavage fluid T cells from COVID-19 patients. **A** The analysis framework of the digitalized sorting of epitope-specific T cells. Epitope-specific T cells were sorted according to their antigenic-specific TCR-β sequence. In this study, influenza A-specific T cell clonotypes were defined by T cell clonotypes with influenza A-specific TCR-β sequence using single-cell RNA sequencing. **B** The donut plot shows the percentage of COVID-19 patients defined by the categories of influenza A-specific T cells and COVID-19 disease severity. The colors represent the COVID-19 patients with or without influenza A-specific T cells in bronchoalveolar lavage fluid T cells. **C** The percentage (%) of influenza A-specific T cells in bronchoalveolar T cells in COVID-19 patients with mild (*n* = 3), severe (*n* = 1), and critical (*n* = 5) disease severity, with color coding according to the specificity of T cells to different epitopes from different genes of influenza A virus. The “*” shows the epitopes that could be found to elicit T cell response

We found that 33.3% of COVID-19 patients had positive anti-influenza T cells in BALF (red group; Fig. 1B). Notably, all patients with positive anti-influenza T cells were patients with critical severity. Comparatively, critical infection was observed in only 33.2% patients with negative anti-influenza T cells (blue group; Fig. 1B). Next, we analyzed the abundance of anti-influenza T cells in BALF from COVID-19 patients and its relationship with disease severity defined by mild, severe, and critical COVID-19 infections. The anti-influenza T cells were defined by different specificities to different epitopes from different genes of the influenza A virus. As shown in Fig. 1C, no anti-influenza T cells could be found in COVID-19 patients with mild and severe infections. Comparatively, patients with critical infection had positive anti-influenza T cells with an average percentage of 1.23% (Fig. 1C). Moreover, it is worth noting that two of eight influenza A-epitopes, GILGFVFTL and SSLENFRAYV, could elicit T cell response in critically severe COVID-19 patients.

The co-infection of COVID-19 with influenza might complicate the diagnosis, treatment, and prognosis of COVID-19, posing potential challenges to public health. In this study, we provide the first evidence in human bronchoalveolar microenvironment that anti-influenza T cells exist in critically severe COVID-19 patients, but not patients with moderate disease severity. The existence of anti-influenza T cells specifically indicates the immunological status for influenza infection. This finding reveals that the co-infection of SARS-CoV-2 with influenza might cause more severe illness, suggesting that the prevention of influenza infection during the current SARS-CoV-2 pandemic might be important for reducing casualties caused by COVID-19. Considering the upcoming flu season will inevitably merge with the current COVID-19 pandemic, influenza vaccination might be beneficial in high‐risk populations of COVID‐19 infection. Additionally, the surveillance of influenza infection should be considered in COVID-19 patients.

## Data Availability

The data that support the findings of this study will be available from the corresponding author upon reasonable request.
